# Author Correction: Epitaxy, exfoliation, and strain-induced magnetism in rippled Heusler membranes

**DOI:** 10.1038/s41467-024-48325-x

**Published:** 2024-05-07

**Authors:** Dongxue Du, Sebastian Manzo, Chenyu Zhang, Vivek Saraswat, Konrad T. Genser, Karin M. Rabe, Paul M. Voyles, Michael S. Arnold, Jason K. Kawasaki

**Affiliations:** 1 Materials Science and Engineering, University of Wisconsin-Madison, Madison, WI USA; 2 Department of Physics and Astronomy, Rutgers University, New Brunswick, NJ USA

Correction to: *Nature Communications* 10.1038/s41467-021-22784-y, published online 03 May 2021

The original version of this Article omitted the following from the Acknowledgements: “Writing and analysis were partially supported by the Air Force Office of Scientific Research (No. FA9550-21-0127)”. This has now been corrected in both the PDF and HTML versions of the Article.

