# Supplementary material for: Epitaxy, exfoliation, and strain-induced magnetism in rippled Heusler membranes
Source: Nat Commun. 2021 May 3;12:2494. doi: 10.1038/s41467-021-22784-y (PMC8093223; doi:10.1038/s41467-021-22784-y)
Supplement: Supplementary file 1 — Supplementary Information [file 41467_2021_22784_MOESM1_ESM.pdf]

Epitaxy, exfoliation, and strain-induced magnetism in rippled Heusler membranes - SUPPLEMENTARY INFORMATION

Dongxue Du,[1] Sebastian Manzo,[1] Chenyu Zhang,[1] Vivek Saraswat,[1] Konrad T. Genser,[2] Karin M. Rabe,[2] Paul M. Voyles,[1] Michael S. Arnold,[1] and Jason K. Kawasaki[1]

[1]*Materials Science and Engineering, University of Wisconsin-Madison, Madison, WI 53706*

[2]*Department of Physics and Astronomy, Rutgers University, New Brunswick, NJ*

(Dated: March 8, 2021)

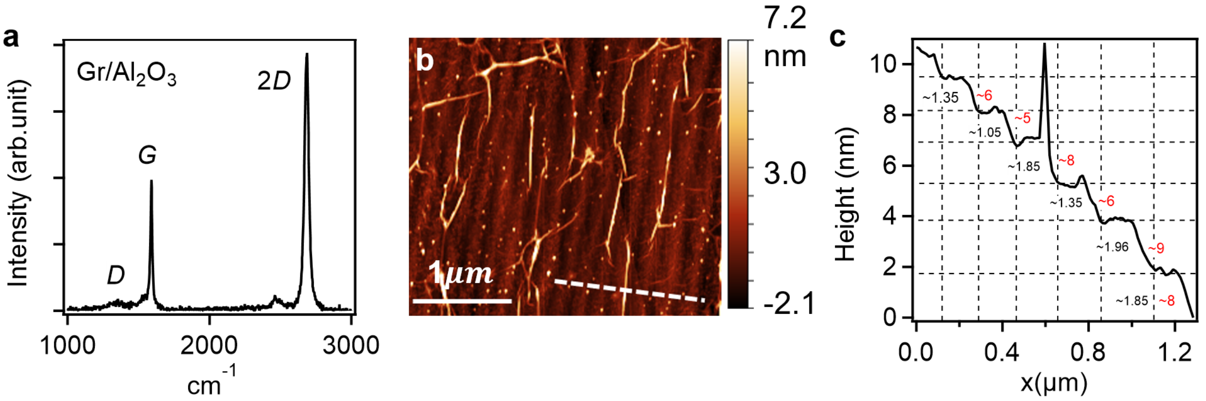

FIG. S-1. Quality of graphene transferred to c-plane sapphire. **a** Raman spectrum of graphene after layer transfer to Al_2O_3. The sharp G and $2D$ modes (FWHM of $2D$-band is $\sim$23 cm^{-1}), and the negligible D-band intensity, indicate a clean transfer with minimal point defects in the graphene [1–4]. Based on small D/G intensity ratio, the average distance between defects is estimated to be $L_D \sim 40nm$ [5]. **b** Atomic force microscopy (AFM) image of graphene on Al_2O_3. We observe a step-and-terrace morphology of the underlying sapphire, along with wrinkles in the graphene that appear as bright streaks. At this length scale we observe no obvious tears; however, we cannot rule out tears or pinholes at a length scale of down to $\sim$ 10 nm, which is typical for CVD-grown graphene [6, 7]. The dashed line marks the position of the AFM line cut in (**c**). The height of each step in (**c**) is labeled by black font and the corresponding number of Al_2O_3 monolayers are labeled by red font. The average step height is 7 monolayers of Al_2O_3.

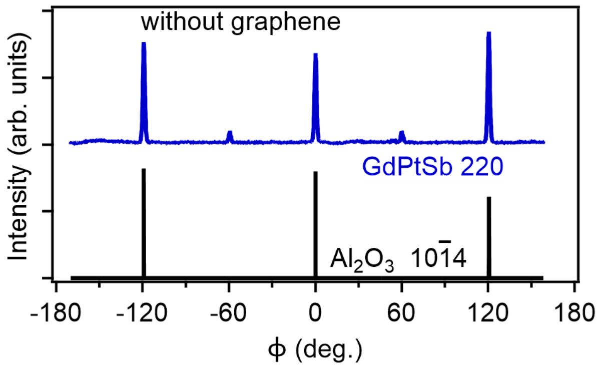

FIG. S-2. Pole figure scans of GdPtSb grown directly on Al_2O_3 without the graphene inter-layer. Only the R0° domain is formed.

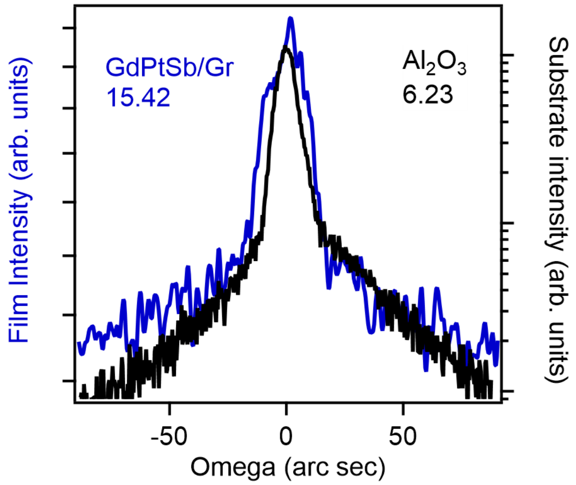

FIG. S-3. XRD rocking curves (Cu $K\alpha$) of GdPtSb on graphene-terminated Al_2O_3 substrate. The black trace is the rocking curve of Al_2O_3 0006 reflection, the blue trace is the rocking curve of GdPtSb 111 reflection. The full width at half maxima (FWHM) for the GdPtSb and Al_2O_3 reflections are 15.42 and 6.23 arc second, respectively.

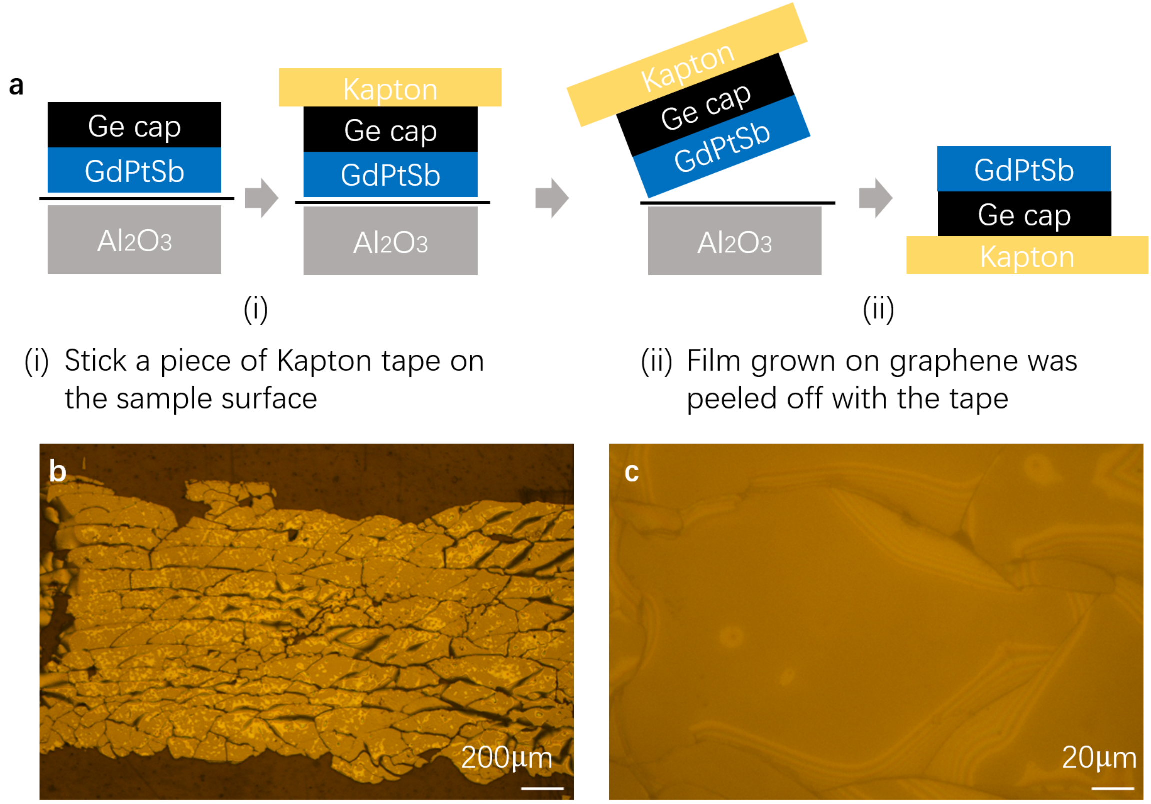

FIG. S-4. Simple exfoliation using Kapton or thermal release tape (TRT). **a** Schematic of the transfer procedure. **b,c** Microscope images of the membrane after exfoliation. The membrane is broken into many small pieces at about 100 μm size. This is caused by the uncontrolled bending of the tape/membrane during exfoliation.

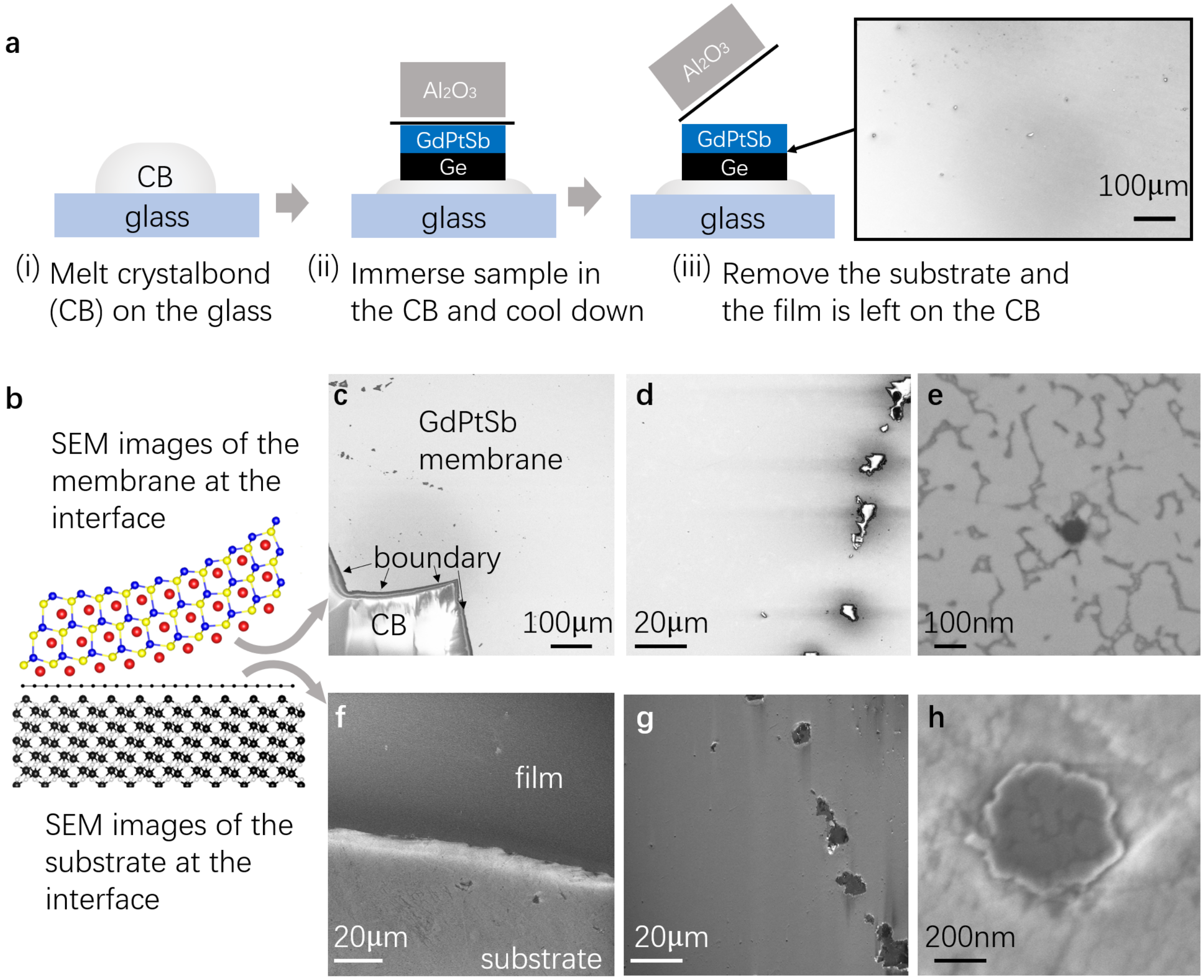

FIG. S-5. Tear-free exfoliation method using Crystalbond. **a** Schematic of the exfoliation process. The sample is bonded film side down onto a rigid glass slide using Ted Pella Crystalbond (CB) 509. Exfoliation is performed by peeling off the substrate, resulting in no observable long-range tears in the SEM image. **b** Cartoon depicting the GdPtSb membrane side and the graphene/sapphire substrate side. **c-e** SEM images of the exfoliated membrane side. **f-h** SEM images of the exposed substrate side. **f** shows the boundary between a region that was covered by graphene and a region where there was no graphene. On the graphene covered region, the GdPtSb membrane could be exfoliated to reveal a bare substrate (lower half of image). On the region with no graphene, the GdPtSb film remains (upper half of image). At the $\sim$ 100 micron scale **c,f** both membrane and substrate side appear uniform indicating a clean exfoliation. At smaller length scales, we observe holes and tears with characteristic length scale $\sim$ 100 nm **e,h** to 10 μm **d,g**.

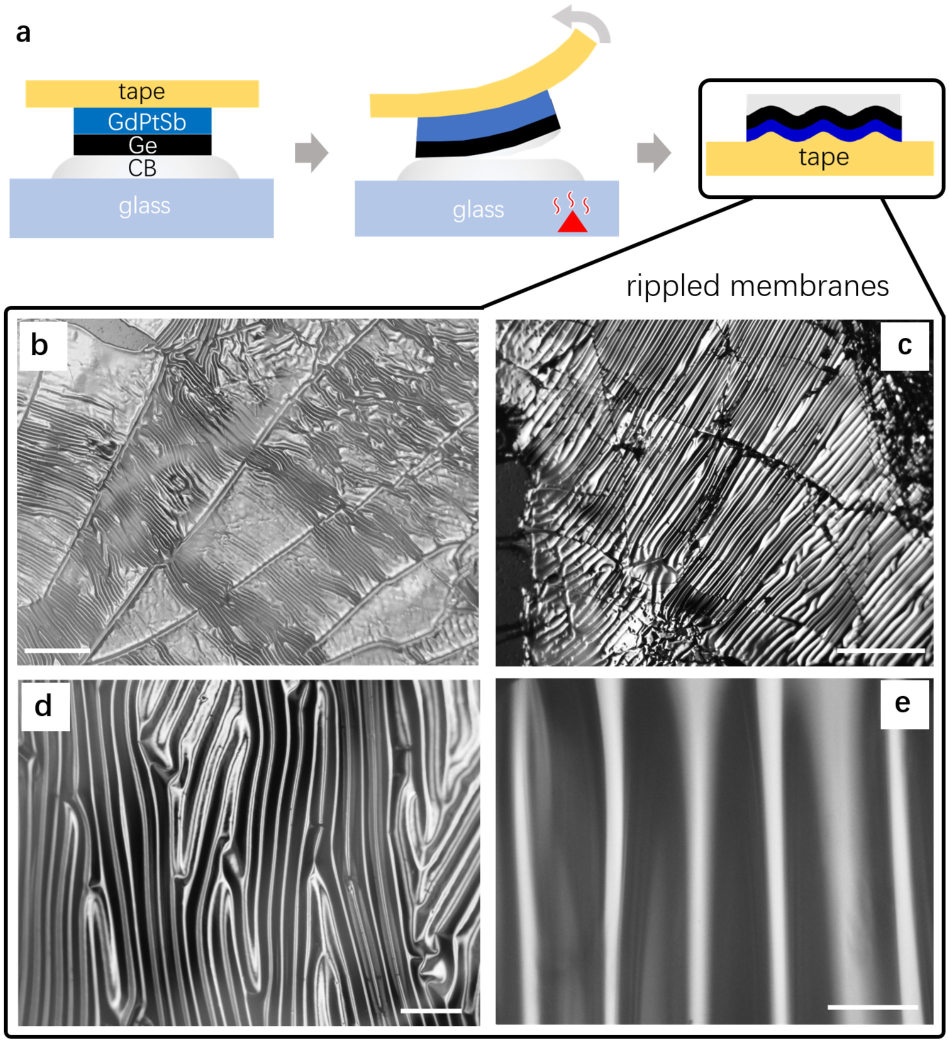

FIG. S-6. Ripple formation. **a** Schematic of the ripple formation process. After exfoliation of the membrane using the glass slide and Crystalbond, the membrane was adhered to a polymer tape (polyurethane, polyimide). Heating the sample on a hot plate melts the crystalbond, allowing the polymer/membrane stack to release. Ripples form during the release and cooldown. Membranes on polymer with larger thermal expansion coefficient have larger ripple aspect ratio. **b-e** Optical microscope images of the rippled membrane on polyimide tape **b,c** and polyurethane tape **d,e**, at various length scales. Scale bars in (**b-e**) are 500μm, 300μm, 100μm and 20μm.

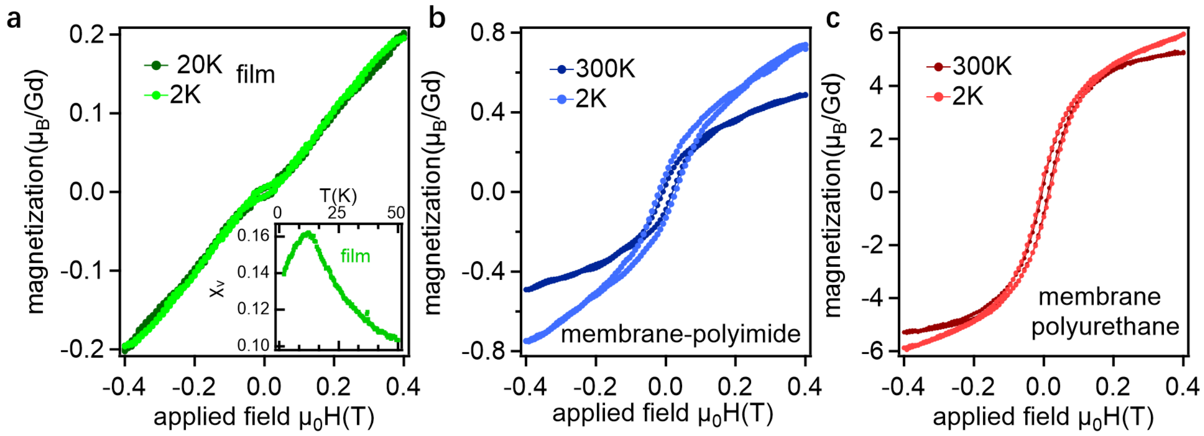

FIG. S-7. Temperature-dependent magnetic properties of GdPtSb films and rippled membranes. **a** The relaxed epitaxial GdPtSb film on sapphire substrate is antiferromagnetic with a Néel temperature of 12 K. **b,c** Ripple strains and strain gradients induce a spontaneous magnetic moment.

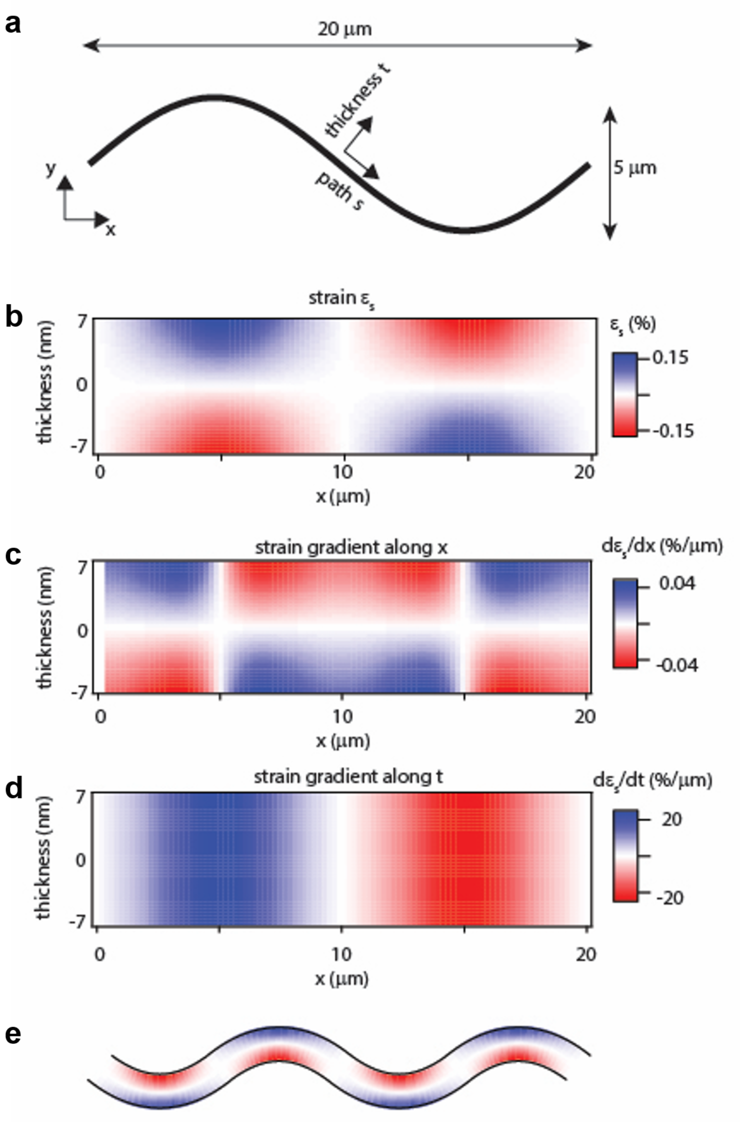

FIG. S-8. Estimation of the strain and strain gradient. The estimate assumes a sinusoidal path $y(x)$ and no plastic deformation. The strain along path s is $\epsilon_s \approx (R+t)/R-1$, where t is the height (or depth) along the thickness axis and the radius of curvature is $R = \left[1 + (y'(x))^2\right]^{3/2} / y''(x)$. **a** Sinusoidal membrane profile with wavelength 20 micron, peak to peak amplitude of 5 micron, and total thickness of 14 nm. **b** Map of the strain along path s. **c** Strain gradient with respect to x: $d\epsilon_s/dx$. **d** Strain gradient along the thickness axis t: $d\epsilon_s/dt$. **e** Strain map projected onto a rippled membrane (schematic).

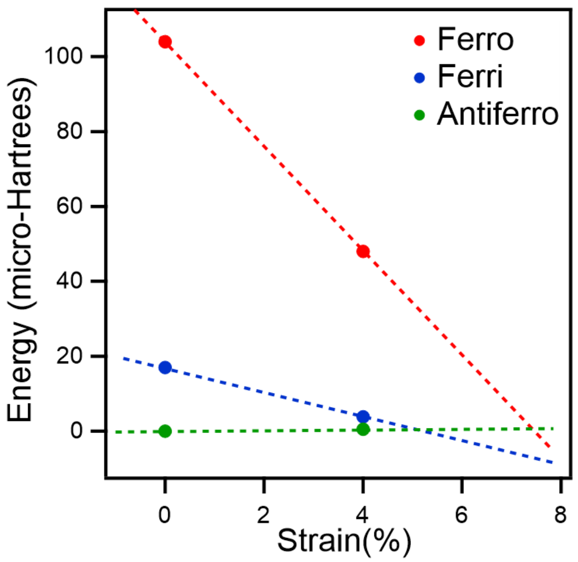

FIG. S-9. First-principles results for the GdPtSb relative total energy as a function of strain, for three different magnetic orderings. The calculations were done on a four-formula-unit supercell. 4% strain corresponds to an expansion by 4% in the (111) plane at fixed volume. Internal structural parameters in fractional coordinates were held fixed at the unstrained value. The calculations were done with spin orbit coupling and magnetization along (111). The input state for the Ferro configuration was all 4 Gd moments pointing along (111). For the Ferri configuration, it was 3 up along (111) and 1 down, and for the Anti-ferro configuration, it was 2 up along (111) and 2 down.

[1] R. Rojas Delgado, R. M. Jacobberger, S. S. Roy, V. S. Mangu, M. S. Arnold, F. Cavallo, and M. G. Lagally, "Passivation of germanium by graphene," *ACS applied materials & interfaces*, vol. 9, no. 20, pp. 17629–17636, 2017.

[2] A. C. Ferrari, J. Meyer, V. Scardaci, C. Casiraghi, M. Lazzeri, F. Mauri, S. Piscanec, D. Jiang, K. Novoselov, S. Roth, *et al.*, "Raman spectrum of graphene and graphene layers," *Physical review letters*, vol. 97, no. 18, p. 187401, 2006.

[3] D. Graf, F. Molitor, K. Ensslin, C. Stampfer, A. Jungen, C. Hierold, and L. Wirtz, "Spatially resolved raman spectroscopy of single-and few-layer graphene," *Nano letters*, vol. 7, no. 2, pp. 238–242, 2007.

[4] L. G. Cançado, A. Jorio, E. M. Ferreira, F. Stavale, C. A. Achete, R. B. Capaz, M. V. d. O. Moutinho, A. Lombardo, T. Kulmala, and A. C. Ferrari, "Quantifying defects in graphene via raman spectroscopy at different excitation energies," *Nano letters*, vol. 11, no. 8, pp. 3190–3196, 2011.

[5] M. M. Lucchese, F. Stavale, E. M. Ferreira, C. Vilani, M. V. d. O. Moutinho, R. B. Capaz, C. A. Achete, and A. Jorio, "Quantifying ion-induced defects and raman relaxation length in graphene," *Carbon*, vol. 48, no. 5, pp. 1592–1597, 2010.

[6] S. C. O'Hern, C. A. Stewart, M. S. Boutilier, J.-C. Idrobo, S. Bhaviripudi, S. K. Das, J. Kong, T. Laoui, M. Atieh, and R. Karnik, "Selective molecular transport through intrinsic defects in a single layer of cvd graphene," *ACS nano*, vol. 6, no. 11, pp. 10130–10138, 2012.

[7] S. S. Roy, R. M. Jacobberger, C. Wan, and M. S. Arnold, "Controlling the density of pinhole defects in monolayer graphene synthesized via chemical vapor deposition on copper," *Carbon*, vol. 100, pp. 1–6, 2016.
